# Secondary Metabolite Profiling, In Vitro Antimicrobial Activity Evaluation, and In Vivo Acute Toxicity Assessment of *Osyris quadripartita* and *Toddalia asiatica* Leaves and Stem Bark Extracts

**DOI:** 10.1155/jotm/8295438

**Published:** 2026-08-07

**Authors:** Eyob Tilahun, Negussie Fedessa Bussa, Anteneh Belayneh, Andarge Zelalem, Tsegu Kiros

**Affiliations:** ^1^ Department of Biotechnology, College of Natural and Computational Science, Wachemo University, Hossana, Ethiopia, wcu.edu.et; ^2^ Department of Food Science and Postharvest Technology, Haramaya Institute of Technology, Haramaya University, P. O. Box 138, Dire Dawa, Ethiopia, haramaya.edu.et; ^3^ School of Biological Sciences and Biotechnology, College of Natural and Computational Sciences, Haramaya University, P. O. Box 138, Dire Dawa, Ethiopia, haramaya.edu.et; ^4^ Chemistry Department, College of Natural and Computational Sciences, Haramaya University, P. O. Box 138, Dire Dawa, Ethiopia, haramaya.edu.et

**Keywords:** acute oral toxicity effect, chloroform extract, methanol extract, *Osyris quadripartita*, secondary metabolites, *Toddalia asiatica*

## Abstract

In Ethiopia, *Osyris quadripartita* (Santalaceae) and *Toddalia asiatica* (Rutaceae) are traditionally used to treat malaria, skin and stomach disorders, and other tropical diseases. This study screened the major secondary metabolite profiles and evaluated the in vitro antimicrobial and in vivo acute toxicity effects of chloroform and methanol extracts from the leaves and stem barks of *O. quadripartita* and *T. asiatica*. Secondary metabolite profiling was conducted according to the standard protocols. The in vitro antimicrobial activity evaluation was performed using Kirby–Bauer and broth dilution assays. The in vivo acute toxicity was assessed in experimental mice, grouped into experimental and control groups. Detected phytochemicals included tannins, alkaloids, saponins, flavonoids, and terpenoids, while phlobatannins were absent. Methanol stem bark extracts of *O. quadripartita* and *T. asiatica* showed higher inhibition zones of 10.67 ± 0.57 and 10.6 ± 0.11 mm, respectively, against methicillin‐resistant *Staphylococcus aureus* at a minimum concentration of 100 mg/mL. These extracts also showed the lowest MIC values against *S. aureus* (6.25 ± 00 mg/mL) and *S. pyogenes* (12.5 ± 00 mg/mL), respectively. The methanol leaf extract of *O. quadripartita* and stem barks of *T. asiatica* demonstrated MBC values of 25 and 50 mg/mL, respectively, against *S. pyogenes*. A higher antifungal effect was observed with the methanol leaf extract of *T. asiatica* against *Aspergillus niger* (8.33 ± 0.28 mm at 100 mg/mL). The present findings revealed that methanol extracts of both plants exhibited better antimicrobial activity than the chloroform extracts, as evidenced by the lowest MIC and MBC values. This aligns with the presence of polar secondary metabolites in methanol extracts, which may be responsible for the observed activity. All crude extracts from both plants showed no signs of toxicity at doses up to 2000 mg/kg in experimental mice, indicating the absence of acute toxicity. In conclusion, these findings support the traditional therapeutic use of the leaves and stem barks of *O. quadripartita* and *T. asiatica* as antimicrobial agents. Further research is needed to quantify the major classes of phytochemicals and identify the compounds responsible for antimicrobial activity using FT‐IR, LC‐MS, and NMR instruments.

## 1. Introduction

Traditional medicinal plants are sources of various biologically active natural molecules that can be used to treat different ailments [1, 2]. The World Health Organization (WHO) describes medicinal plants as the richest sources for exploring numerous medicinally valuable chemical substances [3, 4]. Approximately 60%–80% of the African population relies heavily on plant‐based agents to address both infectious and noninfectious diseases [5–7]. In Ethiopia, medicinal plants also play an indispensable role in the traditional medical system due to their cultural acceptability, economic feasibility, psychological comfort, and trust in their therapeutic effectiveness [8].

*Osyris quadripartita* Decn. (Santalaceae), commonly known as African Sandalwood and synonymous with *O. abyssinica,* is a much‐branched evergreen shrub widely found growing wild at 1500‐2300 m. a.s.l in Ethiopia and other parts of Africa [9]. The plant is indigenous to Africa and grows in gallery forests, *Juniperus, Combretum* and *Dodonaea* woodland, as well as on rocky slopes, degraded woodland, and scrublands [10]. It is known for its fragrant wood, which is used in perfumes and traditional medicine [11]. *O. quadripartita* is among the most frequently consumed medicinally important plants in Ethiopia for the treatment of malaria [12, 13]. Additionally, the plant is used to treat the evil eye, epilepsy, and sexually transmitted diseases (STDs), as an anaphylactic agent [14], and for skin injury, eczema [15], and cancer [16, 17]. Furthermore, *O. quadripartita* is widely applied in the treatment of human ailments such as diarrhea, malaria, fungal infections, and kidney diseases [18, 19]. The powdered roots and leaves of *O. quadripartita* are also used against cancer [20, 21].

*Toddalia asiatica* Lam. (Rutaceae), also known as forest pepper or wild orange tree, is a woody vine (liana) that grows in forests near rivers or streams and occurs in different parts of the world. It is extremely aromatic, releasing a lemon‐like scent when crushed [9]. In East Africa, *T. asiatica* is traditionally used to treat stomach problems, malaria, and other tropical diseases [15]. Pharmacological reports indicate that *T. asiatica* contains bioactive compounds with antibacterial, antiviral, antifungal, anticancer, and antimalarial properties. It has also been reported to have potential against rheumatic discomfort, tumors, knife wound hemorrhage, and traumatic injuries [22].

Human pathogenic microbes such as *Salmonella*, *Escherichia coli* (*E. coli*), *Vibrio cholerae*, and *Shigella* spp. are major causes of diarrhea in children under 5 years of age [23]. The resistance acquired by human pathogenic microbes against currently available antibiotics poses a serious threat to the global healthcare system [24]. Pathogens such as methicillin‐resistant *S. aureus* are a primary focus of drug discovery since they can adapt to various circumstances, thereby causing diverse infections that endanger many lives [25]. Furthermore, *S. pyogenes* is among the human pathogens responsible for skin problems [26].

Taking all these facts into consideration, there is a global call to explore effective, novel, and safe antibiotics [27]. Natural antimicrobial agents derived from medicinal plants are at the forefront of responding to such calls [28]. Although studies have been conducted on *O. quadripartita* and *T. asiatica* in different parts of the world, plants of the same species from different regions can produce secondary metabolites with varied qualitative and quantitative profiles, depending on climate, soil type, nutrient availability, and biotic stresses [29]. These factors directly influence antimicrobial potency. Consequently, regional comparisons are essential for understanding how environmental conditions shape bioactive compound production and give rise to unique antimicrobial chemotypes. Therefore, further investigation of their phytochemical profiles, antimicrobial activities, and toxicological properties through both in vitro and in vivo experiments is still needed. This research was designed to screen the profile of secondary metabolites, evaluate in vitro antimicrobial activity, and assess the in vivo acute oral toxicity properties of chloroform and methanol extracts of *O. quadripartita* and *T*. *asiatica* leaves and stem barks.

## 2. Materials and Methods

### 2.1. Plant Collection and Identification

**FIGURE 1:**
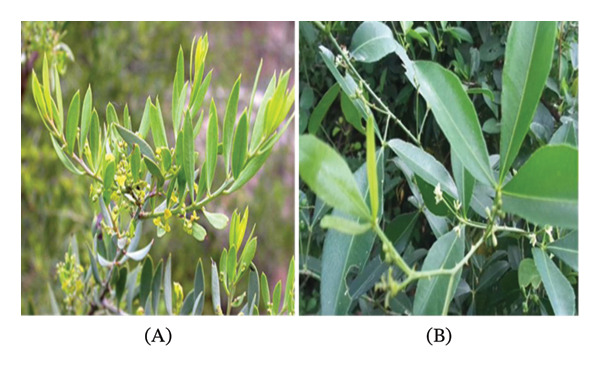
Pictures of *Osyris quadripartita* Decn. (A) and *Toddalia asiatica* (B) (photo courtesy of Anteneh B., December 2024).

**FIGURE 2:**
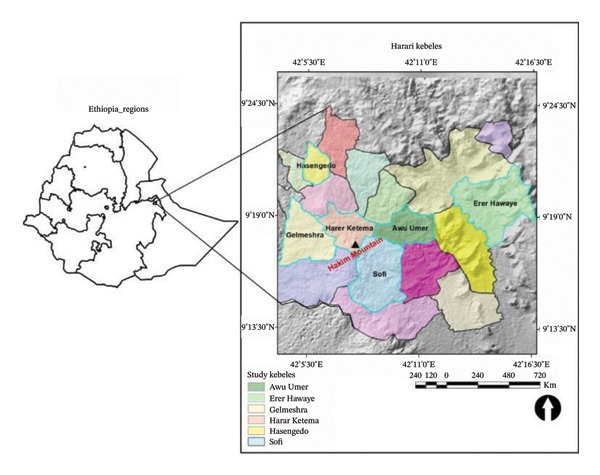
Map of the study area with indication of Hakim Mountain, locally called “Hakim Gara,” where two wild plant species, *Osyris quadripartita* Decn. and *Toddalia asiatica*, were collected (*source*: Anteneh Belayneh—co‐author).

Healthy leaves and stem barks of *O. quadripartita* and *T. asiatica* (Figure 1) were harvested in December 2024 from Hakim Mountain, locally called “Hakim Gara,” at an elevation of 2200 m a.s.l, near the historic city of Harar in Eastern Ethiopia, located 515 km from Addis Ababa, the capital of Ethiopia at 9^o^18′35″ N latitude and 42^o^7′38″ E longitude (Figure 2). Specimens of each plant were brought and identified by a botanist at Haramaya University (Anteneh Belayneh, PhD), using the taxonomic keys of Flora of Ethiopia and Eritrea [9]. The voucher specimens (AHU210 and AHU86, respectively) of both plant species were deposited at the Herbarium of Haramaya University (https://sweetgum.nybg.org/science‐Code ACD).

The study area where the two plant species were collected has an average annual temperature of 19.3°C and an average annual rainfall of 669 mm. There is high variability in annual rainfall, ranging between 275 and 1000 mm. The rainfall pattern of the area is bimodal, occurring from February to April (short rainy season) and June to August (long rainy season) [30].

### 2.2. Experimental Design

The overall antibacterial and antifungal activity tests of the chloroform and methanol extracts of the studied plants were conducted using the paper disc‐diffusion method, while the broth dilution technique was employed to determine the minimum inhibitory concentration (MIC) values. The experimental design for the general in vitro antimicrobial activity was based on a CRD factorial of 4 × 2 × 2 × 2 × 7 × 3 × 3 (four extract concentration levels of 100, 125, 150 and 175 mg/mL; two solvent systems—methanol and chloroform; two sources of extracts—leaves and stem barks; two plant species—*O. quadripartita* and *T. asiatica*; four bacterial and three fungal species; and two positive and one negative controls, with three replications, resulting in 2016 treatment combinations). Ciprofloxacin and ketoconazole/tilt were used as the standard antibiotics for antibacterial and antifungal activity tests, respectively. Dimethyl sulfoxide (4% DMSO) served as the negative control for both experiments. The experimental design applied for the MIC and minimum bactericidal concentration (MBC) against bacterial species was 5 × 2 × 2 × 2 × 4 × 3 (five concentrations of 6.25, 12.5, 25, 50, and 100 mg/mL; two extraction solvents; two plant parts; two plant species; and four bacterial species, with three replications, resulting in 480 total treatments). The experimental design used for the MIC and minimum fungicidal concentration (MFC) against fungal species was 5 × 2 × 2 × 2 × 3 × 3 (five concentrations of 15.625, 31.25, 62.5, 125, and 250 mg/mL; two extraction solvents; two plant parts; two plant species; and three fungal species, with three replications, resulting in 360 total treatments). For the acute oral toxicity assessment, a total of 25 groups (each consisting of six mice) were used (three concentrations of 1000, 1500, and 2000 mg/kg × two plant species × two plant parts × two extraction solvents, plus one negative control group with distilled water), making a total of 150 mice.

### 2.3. Extraction of Plant Samples

Dry powder of leaves and stem barks (100 g each) of *O. quadripartita* and *T. asiatica* was extracted separately using chloroform and methanol (500 mL each) by shaking for 72 h on an orbital shaker through the maceration technique. Each extract solution was then filtered using a suction filtration apparatus, concentrated with a rotary evaporator (rotary vacuum, Jainsons India) at 50°C, and the dried crude extracts were preserved in a refrigerator (4°C) until further use. In this study, chloroform and methanol were selected as extraction solvents to target both nonpolar and polar chemical substances. The maceration extraction technique was chosen because it is simple, requires readily available apparatus, and is suitable for extracting thermolabile chemical constituents.

### 2.4. Qualitative Profiling of Secondary Metabolites

The chloroform and methanol extracts of each plant part were subjected to qualitative secondary metabolite profiling to determine the presence or absence of alkaloids, flavonoids, saponins, steroids, tannins, terpenoids, phenolics, and phlobatannins using the standard method [31].

### 2.5. In Vitro Antimicrobial Activity Study

The in vitro antimicrobial potential of chloroform and methanol extracts of each plant part was evaluated against four bacteria, viz., *S. aureus* (ATCC 25923), *S. pyogenes* (ATCC 196151), *Salmonella Typhi* (*Sa. typhi*, ATCC 13311), and *E. coli* (O157:H7), as well as three pathogenic fungi, namely, *Aspergillus niger* (*A. niger*, ATCC 16888), *A. versicolor* (*A. versicolor*, ATCC 14110), and *Candida albicans* (*C. albicans*, ATCC 10231). All microbial strains were obtained from the Ethiopian Public Health Institute (EPHI), Addis Ababa, Ethiopia. The antimicrobial assay was performed following the Kirby–Bauer disk diffusion procedure outlined by the Clinical and Laboratory Standards Institute (CLSI) [32]. In brief, all test bacteria were subcultured from the original culture (stored at −20°C on plates containing nutrient agar) and incubated at 37°C for 24 h. A few uniform colonies were then aseptically mixed with saline solution, and their turbidity was adjusted to 10^8^ cells/mL according to the 0.5 McFarland standard [33]. Fungal inocula were prepared by taking pure fungal isolates from 48‐hr‐old cultures and mixing them with potato dextrose broth (PDB). The spore density of each fungal culture was adjusted to 10^6^ spores/mL using a spectrophotometer (at 595 nm wavelength) [34]. Stock solutions (200 mg/mL) of each test extract were prepared by dissolving 2 g of the corresponding dry crude extract in 10 mL of 4% DMSO. Four different concentrations (100, 125, 150, and 175 mg/mL) of each extract were then derived from the respective stock solutions [35].

The suspension of each standardized bacterial and fungal inoculum was uniformly swabbed onto Mueller–Hinton agar (MHA, BD) and potato dextrose agar (PDA) plates, respectively. Then, 100 μL of each dilution was loaded onto sterilized Whatman No. 1 filter paper discs (6 mm in diameter), and each disc was placed on the microbial swabbed agar plates, followed by incubation for 24 h at 37°C for the bacterial species and 48 h at 37°C for the fungal species. The experiment was performed in triplicate, and the antimicrobial potential of the extracts was monitored by measuring the diameter of the clear zone (mm) using a digital caliper. Commercial ciprofloxacin (CIP, 5 μg) and ketoconazole/tilt (10 units/disc) served as positive controls for antibacterial and antifungal activity, respectively. DMSO‐impregnated discs were used as negative controls for both antimicrobial activity tests.

### 2.6. MIC Determination

The crude extracts that exhibited in vitro antimicrobial activity were selected to determine the MIC values following the procedure described elsewhere [34]. Five different concentrations (100, 50, 25, 12.5, and 6.25 mg/mL) were prepared by twofold serial dilution, and a nutrient broth (2 mL) was added to each of five test tubes containing 0.1 mL of the respective extract dilutions. After proper mixing, standardized bacterial inoculum (100 μL) was dispensed into the test tubes, which were then incubated at 37°C for 24 h. Similarly, Sabouraud dextrose broth (2 mL) was added to each of five test tubes containing 0.1 mL of crude extracts at different concentrations (250, 125, 62.5, 31.25, and 15.625 mg/mL), and a standardized inoculum (100 μL) of each fungal pathogen was dispensed into the test tubes. These were incubated at 37°C for 48 h. All experiments were performed in triplicate for all test pathogens. Microbial growth after incubation was monitored by comparing the turbidity of the cultures containing the extracts with that of the negative control. The lowest concentration that showed no visible microbial growth was considered the MIC value.

### 2.7. MBC Determination

The MBC value was determined by streaking a loopful of culture from the MIC broth onto nutrient agar and incubating at 37°C for 24 h. The lowest dilution with no growth on the plates was regarded as the MBC.

### 2.8. MFC Determination

The MFC value was determined by streaking a loopful of fungal culture from the MIC broth onto PDA and incubating at 37°C for 48 h. The minimum concentration with no growth on the plates was considered the MFC.

### 2.9. Acute Toxicity Assessment

A total of 150 healthy experimental Swiss albino male and female mice, weighing 28–32 g and aged 6–8 weeks, were used for toxicity evaluation following OECD Guideline 425 [36].

All groups were maintained under 12‐h light and dark cycles at ambient room temperature (22°C–25°C) in the toxicology laboratory of Haramaya University, with free access to a standard rodent pellet diet (from Addis Ababa rodent feed preparation PLC) and water ad libitum. Before the initiation of the experiment, the animals were acclimatized to the laboratory conditions for 1 week [37]. The mice were fasted overnight, and extracts were administrated orally via oro‐gastric cannula. As stated in the experimental design, the total number of groups used was six. Mice in Group 1 were administered 1000 mg/kg of crude extract; Group 2 received 1500 mg/kg; and Group 3 received 2000 gm/kg. The fourth group, serving as the control, was treated with 0.2 mL of distilled water. The animals were observed for physical signs of toxicity for a period of 24 h. Additionally, changes in skin and fur, eyes and mucous membranes, somatomotor activities, and behavioral pattern were monitored. The animals were further observed for convulsions, colonic spasms, salivation, tremors, diarrhea, sleep, coma, and death over a 2‐week period [37].

### 2.10. Statistical Data Analysis

The obtained antimicrobial activity data were statistically evaluated using SPSS statistical software (Version 20) and presented as mean ± standard deviation (SD). The mean values were compared using one‐way analysis of variance (ANOVA), and a *p* value of less than 0.05 was considered to indicate a significant difference between the mean values.

## 3. Results and Discussion

### 3.1. Qualitative Profiling of Secondary Metabolites

The various classes of phytochemicals screened in chloroform and methanol extracts of leaves and stem barks of *O. quadripartita* and *T. asiatica* are presented in Table 1. Both extracts from the two plant parts revealed a complete absence of phlobatannins. The chloroform extracts of both plants’ parts also showed no presence of saponins. On the other hand, alkaloids, flavonoids, saponins, terpenoids, and tannins were positively detected in both extracts of both parts of the two studied plants. Other studies on qualitative phytochemical screening of the methanol extract of *T. asiatica* leaves also reported the presence of alkaloids, flavonoids, steroids, saponins, terpenoids, and tannins [38, 39], which was in line with our results, except for the steroids, which were negatively detected herein. The *T. asiatica* plant has also been reported for its content of important secondary metabolites such as flavonoids, saponins, coumarone, steroids, and alkaloids [40]. The variation in the levels of phytochemicals within the same plant species could be attributed to several factors, including geographical and seasonal variation and method of extraction [41]. In general, plants of the same species from different regions can produce varying types and concentrations of secondary metabolites depending on climate, soil type and nutrient availability, and biotic stresses [29].

**TABLE 1:** Phytochemical analysis of methanol and chloroform extracts of leaves and stem barks of *Osyris quadripartita* and *Toddalia asiatica*.

Solvent extracts	Phytochemicals	Test methods	*O. quadripartita*		*T. asiatica*	
			Leaves	Stem barks	Leaves	Stem barks
Methanol	Alkaloids	Dragendorff	+	+	+	+
	Flavonoids	Ammonium	+	+	+	+
	Saponins	Froth	+	+	+	+
	Steroids	Liebermann–Burchard	+	+	−	+
	Tannins	Chloride	+	+	+	+
	Terpenoids	Salkowski	+	+	+	+
	Phenolic compounds	Riberiero	+	+	+	−
	Phlobatannins	Shaik	−	−	−	−

Chloroform	Alkaloids	Dragendorff	+	+	−	+
	Flavonoids	Ammonium	+	+	+	+
	Saponins	Froth	−	−	−	−
	Steroids	Liebermann–Burchard	+	+	−	−
	Tannins	Chloride	+	+	+	+
	Terpenoids	Salkowski	+	−	+	+
	Phenolic compounds	Riberiero	+	+	−	−
	Phlobatannins	Shaik	−	−	−	−

*Note:* Positive (+) sign indicates the presence of the phytochemical compounds, while negative (−) sign indicates the absence of phytochemical compounds.

### 3.2. In Vitro Antimicrobial Activity

The observed in vitro antimicrobial activities, expressed as the diameter of the zone of inhibition (mm), against four bacterial and three fungal strains tested with methanol and chloroform extracts of the leaves and stem barks of *O. quadripartita* and *T. asiatica* are presented in Tables 2 and 3, respectively. It is important to note that, during the interpretation of inhibition zone values of plant extracts, there is no universally standardized breakpoint, unlike the one established by the Clinical and Laboratory Standards Institute for antibiotics. However, according to Eloff [42], a Section Editor of BMC Complementary and Alternative Medicine, the antimicrobial activity of plant extracts can be interpreted based on commonly used empirical criteria reported in several previous phytochemical studies, where inhibition zones ≥ 15 mm indicate strong activity, 10–14 mm moderate activity, and < 10 mm weak activity. Based on this explanation, the methanol leaf extracts of *O. quadripartita* and *T. asiatica* displayed a weak‐to‐moderate antibacterial activity against *E. coli*, *S. aureus*, and *S. pyogenes* at all concentrations, with inhibitory values ranging from 7.67 ± 0.57 to 13.4 ± 0.52 mm and 8.06 ± 0.11 to 13.33 ± 0.57 mm, respectively. On the other hand, the methanol stem bark extracts of both plants showed weak‐to‐strong activity against *S. aureus*, with inhibitory values ranging from 8.26 ± 0.25 to15.23 ± 0.25 mm, and weak‐to‐moderate activity against S*. pyogenes* (inhibitory values of 8.67 ± 0.57 to 13.06 ± 0.11 mm) at all concentrations. The *S. typhi* bacterial strain was found resistant to all methanol extracts of both plants, whereas *E. coli* showed resistance to the methanol stem bark extracts of both plants (Table 2). The chloroform extracts of the leaves and stem barks of *O. quadripartita* were found to be weakly and moderately active against *S. aureus* and *S. pyogenes* (8.33 ± 0.75 to 11.67 ± 0.56 and 9.16 ± 0.28 to 12.16 ± 0.15, respectively) at all concentrations (Table 3). Similarly, the chloroform extracts of the leaves and stem barks of *T. asiatica* showed weak‐to‐moderate activity against *S. aureus* and *S. pyogenes*, with inhibitory values of 7.33 ± 0.28 to11.73 ± 0.46 mm and 9.16 ± 0.28 to 12.67 ± 0.57 mm, respectively, at all dilutions (Table 3). However, the chloroform extracts of both parts of both plants exhibited complete resistance from *E. coli* and *S. typhi*. The methanol extract of the leaves of *O. quadripartita* displayed weak activity (9.83 ± 0.23 mm) against *S. pyogenes,* while the methanol extract of the stem barks of the same plant displayed a moderate inhibitory value (10.67 ± 0.57 mm) against *S. aureus* at the lowest concentration of 100 mg/mL. Similarly, the methanol extracts of both the leaves and stem barks of *T. asiatica* indicated weak and moderate inhibitory potential (9.83 ± 0.28 and 10.6 ± 0.11 mm, respectively) against *S. aureus* at the lowest concentration (100 mg/mL). This observation aligns with previous report indicating that methanolic plant extracts have generally demonstrated better antibacterial potency against Gram‐positive bacteria than Gram‐negative ones [43], due to the differences in the biological makeup of the pathogens, in which Gram‐negative bacteria possess an extra lipid outer membrane that creates a permeability barrier to polar compounds. Among the chloroform extracts, the stem bark of *T. asiatica* exhibited a weak antibacterial effect (9.76 ± 0.04 mm) against *S. pyogenes*, followed by the stem bark of *O. quadripartita* (9.67 ± 0.57 mm) against *S. aureus* at the lowest concentration (100 mg/mL). The *S. typhi* bacterial strain was found to be resistant to all tested extracts, while *E.coli* showed total resistance to the methanol stem bark extracts and all chloroform extracts of both plants at all concentrations. The standard antibacterial drug, ciprofloxacin, recorded inhibitory values of 16.33 ± 9.57 to 18 ± 1 mm against the tested bacterial strains. In general, the antibacterial activity of the methanol extracts of leaves and stem barks of *O. quadripartita* and *T. asiatica* was found to be comparable to results reported elsewhere [44].

**TABLE 2:** In vitro antimicrobial inhibitory values (mean ± standard deviation) of methanol extracts of leaves and stem barks of *Osyris quadripartita* and *Toddalia asiatica* against selected bacterial and fungal strains.

Tested plant/plant part	Concentration (mg/mL)	Zone of inhibition values (mean ± standard deviation) against tested microbial species						
		Bacterial species				Fungal species		
		*E. coli*	*S. typhi*	*S. aureus*	*S. pyogenes*	*C. albicans*	*A. niger*	*A. versicolor*
*O. quadripartita/*leaves	100	7.67 ± 0.57^Bb^	−	8 ± 0.5^Ca^	9.83 ± 0.23^Db^	7.16 ± 0.28^Ba^	7.33 ± 0.57^Bb^	7.23 ± 0.25^Bb^
	125	8.4 ± 0.17^Cc^	−	8.83 ± 0.28^Ca^	11.06 ± 0.12^Dd^	7.67 ± 0.28^Ba^	7.67 ± 0.57^Bb^	7.83 ± 0.28^Bb^
	150	8.67 ± 0.57^Cc^	−	9.6 ± 0.57^Eb^	11.76 ± 0.4^Ef^	8.33 ± 0.28^Bbc^	9.16 ± 0.28^Dde^	8 ± 0.6^Bc^
	175	9.67 ± 0.57^Ce^	−	11.83 ± 0.28^De^	13.4 ± 0.52^Df^	8.67 ± 0.57^Bc^	10.33 ± 0.57^Df^	9.13 ± 0.23^Bd^

*O. quadripartita*/stem barks	100	−		10.67 ± 0.57^Dd^	8.26 ± 0.25^Ca^	7.33 ± 0.28^Ba^	−	−
	125	−		11.67 ± 0.57^Df^	9.8 ± 0.34^Cb^	8.33 ± 0.57^Bb^	−	−
	150	−		13.33 ± 0.57^Dg^	11.83 ± 0.28^Cd^	9.16 ± 0.28^Bc^	−	−
	175	−		15.23 ± 0.25^Di^	14.33 ± 0.57^Cg^	10.33 ± 0.57^Bf^	−	−

*T. asiatica*/leaves	100	8.67 ± 0.57^Cc^	−	9.83 ± 0.28^Dc^	8.06 ± 0.11^Ba^	7.67 ± 0.57^Ba^	8.33 ± 0.57^Cc^	−
	125	9.33 ± 0.28^Cd^	−	10.8 ± 0.52^De^	9.33 ± 0.57^Ca^	8.67 ± 0.28^Bc^	8.33 ± 0.28^Bd^	−
	150	9.67 ± 0.57^Ce^	−	11.7 ± 0.60^Dg^	10.13 ± 0.11^Cb^	8.83 ± 0.28^Bc^	8.6 ± 0.57^Bd^	−
	175	10.3 ± 0.28^Cf^	−	13.33 ± 0.57^Eh^	11.5 ± 0.86^Dd^	9.83 ± 0.28^Ce^	9.33 ± 0.28^Be^	−

*T. asiatica*/stem barks	100	−	−	10.6 ± 0.11^Dc^	8.67 ± 0.57^Ca^	7.16 ± 0.28^Ba^	−	−
	125	−	−	10.5 ± 0.86^Dc^	9.66 ± 0.57^Cb^	7.16 ± 0.28^Ba^	−	−
	150	−	−	11.66 ± 0.57^Dg^	10.83 ± 0.28^Cc^	8.5 ± 0.5^Bb^	−	−
	175	−	−	13.06 ± 0.11^Ch^	13.06 ± 0.11^Cf^	9.33 ± 0.57^Be^	−	−

Ciprofloxacin	5 μg/disc	18 ± 1.0^Bg^	17 ± 1.0^Ab^	16.33 ± 0.57^Aj^	16.33 ± 0.57^Ah^			

Ketoconazole/tilt	100 units/disc					18 ± 1.0^Bg^	17.67 ± 0.57^Ag^	17 ± 1.0^Be^

DMSO (4%)		−	−	−	−	−	−	−

*Note:* Uppercase letters compare the means between the rows against concentrations, and lowercase letters compare the means between the columns against microbes. Means with different uppercase and lowercase letters are significantly different values (*p* < 0.05). Negative (−) sign indicates that no inhibitory effect was observed. Inhibition zones (mean ± standard deviation in mm) ≥ 15 mm indicate a strong activity, 10–14 mm moderate activity, and < 10 mm weak activity.

**TABLE 3:** In vitro antimicrobial inhibitory values (mean ± standard deviation) of chloroform extracts of leaves and stem barks of *Osyris quadripartita* and *Toddalia asiatica*.

Tested plant/plant part	Concentration (mg/mL)	Zone of inhibition values (mean ± standard deviation) against tested microbial species						
		Bacterial species				Fungal species		
		*E. coli*	*S. typhi*	*S. aureus*	*S. pyogenes*	*C. albicans*	*A. niger*	*A. versicolor*
*O. quadripartita*/leaves	100	−	−	8.6 ± 0.57^Ca^	8.33 ± 0.57^Cb^	−	7.33 ± 0.57^Bb^	7.23 ± 0.25^Bb^
	125	−	−	9.83 ± 0.28^Dc^	9.33 ± 0.57^Cc^	−	8.13 ± 0.28^Bc^	7.33 ± 0.28^Bb^
	150	−	−	10.8 ± 0.52^Be^	9.6 ± 0.57^Ccd^	−	9.16 ± 0.28^Cde^	7.833 ± 0.28^Bcd^
	175	−	−	11.67 ± 0.56^Df^	10.83 ± 0.28^Ce^	−	10.33 ± 0.57^Ce^	8.83 ± 0.28^Bd^

*O. quadripartita*/stem barks	100	−	−	9.67 ± 0.57^Db^	9.16 ± 0.28^Cc^	−	−	8.16 ± 0.28^Bc^
	125	−	−	11.33 ± 0.57^Ce^	11.13 ± 0.23^Cd^	−	−	8.67 ± 0.57^Bd^
	150	−	−	11.4 ± 0.52^Ce^	11.6 ± 0.57^cd^	−	−	10.0 ± 0.5^Be^
	175	−	−	12.3 ± 0.28^Cg^	12.16 ± 0.15^ce^	−	−	10.67 ± 0.57^Bef^

*T. asiatica*/leaves	100	−	−	9.06 ± 0.12^Ca^	7.33 ± 0.28^Ba^	−	−	−
	125	−	−	9.93 ± 0.40^Cd^	8.13 ± 0.12^Bb^	−	−	−
	150	−	−	10.73 ± 0.46^Bc^	9.16 ± 0.28^Bc^	−	−	−
	175	−	−	11.73 ± 0.46^Cg^	9.73 ± 0.46^Bc^		−	−

*T. asiatica* stem/barks	100	−	−	9.16 ± 0.28^Ca^	9.76 ± 0.04^Dc^	8.16 ± 0.28^Bb^	7.67 ± 0.57^Bb^	−
	125	−	−	9.73 ± 0.46^Bb^	11.76 ± 0.46^Cd^	10.16 ± 0.28^Bc^	10.33 ± 0.57^Bd^	−
	150	−	−	10.73 ± 0.46^Bd^	11.83 ± 0.28^Ce^	9.66 ± 0.57^Bd^	9.5 ± 0.86^Be^	−
	175	−	−	12.06 ± 0.12^Cg^	12.67 ± 0.57^Df^	11.17 ± 0.28^Be^	10.83 ± 0.28^Be^	−

Ciprofloxacin	5 μg/disc	18 ± 1.0^Cb^	17 ± 1.0^Bb^	16.33 ± 0.57^Bh^	16.33 ± 0.57^Bh^			

Ketoconazole/tilt	100 units/disc					18 ± 1.0^Cf^	17.66 ± 0.57^Cf^	17 ± 1.0^Bg^

DMSO (4%)		−	−	−	−	−	−	−

*Note:* Uppercase letters compare the means between the rows against concentrations, and lowercase letters compare the means between the columns against microbes. Means with different upper and lowercase letters are significantly different values (*p* < 0.05). Negative (−) sign indicates that no inhibitory effect was observed. Inhibition zones (mean ± standard deviation in mm) ≥ 15 mm indicate a strong activity, 10–14 mm moderate activity, and < 10 mm weak activity.

The antifungal activity study demonstrated that the methanol leaf extract of *O. quadripartita* showed a weak‐to‐moderate inhibitory effect on the growth of all tested fungal strains, with inhibitory values ranging from 7.16 ± 0.28 to 10.33 ± 0.57 mm, while the methanol extract of the stem barks of the same plant failed to inhibit the growth of *A. niger* and *A. versicolor* at all tested doses (Table 2). The methanol extract of the leaves of *T. asiatica* was found to be weakly active against the *C. albicans* and *A. niger* strains, with zones of inhibition ranging from 7.67 ± 0.57 to 9.33 ± 0.28 mm, while it was completely inactive against *A. versicolor* at all concentrations. On the other hand, the methanol extract of the stem barks of *T. asiatica* did not show activity against *A. niger* and *A. versicolor*, but weakly inhibited the growth of *C. albicans*, with inhibition zones ranging from 7.16 ± 0.28 to 9.33 ± 0.57 mm at all concentrations (Table 2). All the tested chloroform extracts were found to be inactive against *C. albicans*, except for the stem barks of *T. asiatica*, which showed a weak‐to‐moderate activity (8.16 ± 0.28 to 11.17 ± 0.28 mm) at all concentrations (Table 3). On the other hand, no activity was observed for the chloroform extract of the stem barks of *O. quadripartita* against *A. niger* and for the chloroform extract of the leaves of *T. asiatica* against *A. niger* and *A. versicolor* at all tested concentrations (Table 3). Weak antifungal activity (8.33 ± 0.28 mm) at the lowest concentration of 100 mg/mL was observed for the methanol extract of the leaves of *T. asiatica* against *A. niger*, followed by the chloroform extracts (8.16 ± 0.28 mm) of the stem barks of *O. quadripartita* and *T. asiatica* against *A. versicolor* and *C. albicans*, respectively. The standard antifungal drug ketoconazole recorded inhibitory values ranging from 17 ± 1 to 18 ± 1 mm against the tested fungal strains. In this study, the insignificant activity of *T. asiatica* against *A. niger* was in agreement with previously reported work [45]. Similarly, relatively weak activity of *T. asiatica* extract against *C. albicans* was previously reported [46]. Overall, the antimicrobial data demonstrated that the methanol extracts exhibited better antimicrobial activity than the chloroform extracts. This enhanced efficacy is consistent with the presence of polar secondary metabolites, such as, alkaloids, steroids, terpenoids, flavonoids, and tannins in methanol extracts, which are likely responsible for the observed activity [47].

### 3.3. Results of MIC, MBC, and MFC

The MIC values of methanol and chloroform extracts from the leaves and stem barks of *O. quadripartita* and *T. asiatica* are presented in Table 4. The MIC values for the methanol and chloroform leaf extracts of *O. quadripartita* ranged between 35.5 ± 13 and 100 ± 00 mg/mL, and 31.25 ± 00 and 250 ± 00 mg/mL, respectively, against bacterial and fungal pathogens. The MIC values for the methanol and chloroform stem bark extracts of the same plant were found to be between 12.5 ± 00 and 100 ± 00 mg/mL against bacterial and 62.5 ± 00 and 250 ± 00 mg/mL against fungal strains. The methanol and chloroform leaf extracts of *T. asiatica* showed MIC values ranging from 25 ± 00 to 100 ± 00 mg/mL and 31.25 ± 00 to 62.5 ± 00 mg/mL against bacterial and fungal species, respectively, whereas the MIC values of the same solvent extracts prepared from the stem barks of the same plant were found to be between 6.25 ± 00 and 100 ± 00 mg/mL, and 62.5 ± 00 and 250 ± 00 mg/mL against bacteria and fungi, respectively. The methanol extracts of the stem barks of both *T. asiatica* and *O. quadripartita* showed the lowest MIC values of 6.25 ± 00 and 12.5 ± 00 mg/mL against methicillin‐resistant *S. aureus* and *S. pyogenes* strains, respectively. The methanol leaf extracts of *T. asiatica* and *O. quadripartita* were found to have the lowest MIC values against the fungus *A. niger* (31.25 ± 00 mg/mL) and the bacterium *S. pyogenes* (35.5 ± 13 mg/mL), respectively. From the chloroform extract groups, the leaves of *T. asiatica* showed the lowest MIC value (25 ± 00 mg/mL) against the Gram‐positive *S. pyogenes*, followed by the leaves of *O. quadripartita* (31.25 ± 00 mg/mL) against the fungus *A. versicolor*.

**TABLE 4:** MIC values (mg/mL) of methanol and chloroform extracts of leaves and stem barks of *Osyris quadripartita* and *Toddalia asiatica* against bacterial and fungal pathogens.

Microbial pathogens	MIC values (mg/mL) of methanol extracts				MIC values (mg/mL) of chloroform extracts			
	*O. quadripartita*		*T. asiatica*		*O. quadripartita*		*T. asiatica*	
	Leaves	Stem barks	Leaves	Stem barks	Leaves	Stem barks	Leaves	Stem barks
*E. coli*	100 ± 00	−	50 ± 00	−	−	−	−	−
*S. typhi*	−	−	—	—	—	—	—	—
*S. aureus*	100 ± 00	100 ± 00	100 ± 00	6.25 ± 00	50 ± 00	80 ± 22.4	50 ± 00	100 ± 00
*S. pyogenes*	35.5 ± 13	12.5 ± 00	100 ± 00	80 ± 22.4	50 ± 00	50 ± 00	25 ± 00	35.5 ± 13
*C. albicans*	125 ± 00	250 ± 00	62.5 ± 00	125 ± 00	—	—	—	250 ± 00
*A. niger*	62.5 ± 00	—	31.25 ± 00	—	—	—	—	62.5 ± 00
*A. versicolor*	250 ± 00	—	—	—	31.25 ± 00	62.5 ± 00	—	—

*Note:* Values are expressed as mean ± SD (*n* = 3). Negative (−) sign indicates that no inhibitory effect was observed at the higher concentration (100 mg/mL). Lower values (mg/mL) across each column and row indicate a higher activity.

The MBC and MFC values of methanol leaf and stem bark extracts of *O. quadripartita* and *T. asiatica* are presented in Tables 5 and 6, respectively, while those of chloroform extracts of the respective plants are shown in Tables 7 and 8. The methanol extract of the leaf of *O. quadripartita* (Table 5) and the stem barks of *T. asiatica* (Table 6) exhibited better activity against *S. pyogenes*, with MBC values of 25 and 50 mg/mL, respectively, while the chloroform stem bark extract of *O. quadripartita* showed bactericidal activity against methicillin‐resistant *S. aureus* at 50 mg/mL (Table 7). On the other hand, all extracts of both parts of the investigated plants did not show fungicidal activity below the maximum concentration (250 mg/mL) against all fungal species.

**TABLE 5:** MBC and MFC values (mg/mL) of methanol extracts of leaves and stem barks of *Osyris quadripartita* against bacterial and fungal pathogens.

Pathogen categories	Leaf					Stem bark				
Bacterial pathogens	Concentrations (mg/mL)									
	100	50	25	12.5	6.25	100	50	25	12.5	6.25
*E. coli*	+	−	−	−	−	−	−	−	−	−
*S. typhi*	−	−	−	−	−	−	−	−	−	−
*S. aureus*	+	−	−	−	−	+	−	−	−	−
*S. pyogenes*	+	+	+	−	−	−	−	−	−	−

**Fungal pathogens**	**250**	**125**	**62.5**	**31.25**	**15.63**	**250**	**125**	**62.5**	**31.25**	**15.63**

*C. albicans*	−	−	−	−	−	+	−	−	−	−
*A. niger*	−	−	−	−	−	−	−	−	−	−
*A. versicolor*	+	−	−	−	−	−	−	−	−	−

*Note:* Values are expressed as mean ± SD (*n* = 3). Negative (−) sign indicates that no bactericidal and/or fungicidal effect was observed, while positive (+) sign indicates the bactericidal and/or fungicidal effect of extracts.

**TABLE 6:** MBC and MFC values (mg/mL) of methanol extracts of leaf and stem bark of *Toddalia asiatica* against bacterial and fungal pathogens.

Pathogen categories	Leaf					Stem bark				
Concentrations (mg/mL)										
Bacterial pathogens	100	50	25	12.5	6.25	100	50	25	12.5	6.25
*E. coli*	+	−	−	−	−	−	−	−	−	−
*S. typhi*	−	−	−	−	−	−	−	−	−	−
*S. aureus*	+	−	−	−	−	−	−	−	−	−
*S .pyogenes*	+	−	−	−	−	+	+	−	−	−

**Fungal pathogens**	**250**	**125**	**62.5**	**31.25**	**15.63**	**250**	**125**	**62.5**	**31.25**	**15.63**

*C. albicans*	−	−	−	−	−	−	−	−	−	−
*A. niger*	−	−	−	−	−	−	−	−	−	−
*A. versicolor*	−	−	−	−	−	−	−	−	−	−

*Note:* Values are expressed as mean ± SD (*n* = 3). Negative (−) sign indicates that no bactericidal and/or fungicidal effect was observed, while positive (+) sign indicates bactericidal and/or fungicidal effect of extracts.

**TABLE 7:** MBC and MFC values (mg/mL) of chloroform extracts of leaves and stem barks of *Osyris quadripartita* against bacterial and fungal pathogens.

Pathogen categories	Leaf					Stem bark				
Concentrations (mg/mL)										
Bacterial pathogens	100	50	25	12.5	6.25	100	50	25	12.5	6.25
*E. coli*	−	−	−	−	−	−	−	−	−	−
*S. typhi*	−	−	−	−	−	−	−	−	−	−
*S. aureus*	−	−	−	−	−	+	+	−	−	−
*S. pyogenes*	−	−	−	−	−	−	−	−	−	−

**Fungal pathogens**	**250**	**125**	**62.5**	**31.25**	**15.625**	**250**	**125**	**62.5**	**31.25**	**15.625**

*C. albicans*	−	−	−	−	−	−	−	−	−	−
*A. niger*	−	−	−	−	−	−	−	−	−	−
*A. versicolor*	−	−	−	−	−	−	−	−	−	−

*Note:* Values are expressed as mean ± SD (*n* = 3). Negative (−) sign indicates that no bactericidal and/or fungicidal effect was observed, while positive (+) sign indicates bactericidal and/or fungicidal effect of extracts.

**TABLE 8:** MBC and MFC values (mg/mL) of chloroform extracts of leaf and stem bark of *Toddalia asiatica* against bacterial and fungal pathogens.

Pathogen categories	Leaf					Stem bark				
Concentrations (mg/mL)										
Bacterial pathogens	100	50	25	12.5	6.25	100	50	25	12.5	6.25
*E. coli*	−	−	−	−	−	−	−	−	−	−
*S. typhi*	−	−	−	−	−	−	−	−	−	−
*S. aureus*	−	−	−	−	−	+	−	−	−	−
*S. pyogenes*	−	−	−	−	−	−	−	−	−	−

**Fungal pathogens**	**250**	**125**	**62.5**	**31.25**	**15.265**	**250**	**125**	**62.5**	**31.25**	**15.625**

*C. albicans*	−	−	−	−	−	+	−	−	−	−
*A. niger*	−	−	−	−	−	−	−	−	−	−
*A. versicolor*	−	−	−	−	−	−	−	−	—	−

*Note:* Values are expressed as mean ± SD (*n* = 3). Negative (−) sign indicates that no bactericidal and/or fungicidal effect was observed, while positive (+) sign indicates bactericidal and/or fungicidal effect of extracts.

The present findings indicate that the methanol extracts of both the leaves and stem barks of the two plants exhibited moderate antibacterial and antifungal activities, with lower MIC and MBC values than the chloroform extracts. This was found to be in agreement with other reports [41, 48].

### 3.4. Result of Oral Acute Toxicity

An in vivo study on the oral acute toxicity effects of methanol and chloroform leaves and stem bark extracts from *O. quadripartita* and *T. asiatica* was carried out in mice (Tables 9, 10, and 11). The results indicated that the aforementioned extracts did not exert any signs of toxic impact on overall physical and behavioral changes, including reduced appetite, hair erection, motor activity, weight loss, lacrimation, depression, abnormal secretion, and diarrhea in the experimental mice when continuously monitored for 24 h at dosages of 1000, 1500, and 2000 mg/kg body weight (Table 9). Moreover, the extracts caused no mortality at 1000, 1500, and 2000 mg/kg within the first 24 h or during the subsequent 14 days. Furthermore, the mice did not show any changes in eyelids, skin color, food intake, sleep, water intake, or other noticeable toxicity symptoms during the 14‐day toxicity test. This highlighted that the metabolic reactions of biomolecules in the mice, including proteins, carbohydrates, and fats, were not disturbed. These toxicity results were consistent with previously reported similar studies [49, 50]. This suggests that the oral median lethal dose (LD) of the extracts could be higher than 2000 mg/kg body weight. These findings most likely justify the use of chloroform and methanol extracts of the leaves and stem barks of *O. quadripartita* and *T. asiatica* for the treatment of microbial pathogens.

**TABLE 9:** Daily behavioral changes observed during acute toxicity studies of methanol and chloroform extracts of leaves and stem barks of *Osyris quadripartita* and *Toddalia asiatica* in Swiss albino mice.

Gross activity	30 min	1 h	2 h	3 h	4 h	24 h	Day	Day	Day	Day	Day	Day	Day	Day	Day	Day	Day	Day	Day
							2	3	4	5	6	7	8	9	10	11	12	13	14
Respiration	+	+	+	+	+	+	+	+	+	+	+	+	+	+	+	+	+	+	+
Writhing	−	−	−	−	−	−	−	−	−	−	−	−	−	−	−	−	−	−	−
Tremor	−	−	−	−	−	−	−	−	−	−	−	−	−	−	−	−	−	−	−
Convulsion	−	−	−	−	−	−	−	−	−	−	−	−	−	−	−	−	−	−	−
Sense of touch/sound	+	+	+	+	+	+	+	+	+	+	+	+	+	+	+	+	+	+	+
Salivation	+	+	+	+	+	+	+	+	+	+	+	+	+	+	+	+	+	+	+
Urination	+	+	+	+	+	+	+	+	+	+	+	+	+	+	+	+	+	+	+
Defecation	+	+	+	+	+	+	+	+	+	+	+	+	+	+	+	+	+	+	+
Diarrhea	−	−	−	−	−	−	−	−	−	−	−	−	−	−	−	−	−	−	−
Sedation	−	−	−	−	−	−	−	−	−	−	−	−	−	−	−	−	−	−	−
Locomotion	+	+	+	+	+	+	+	+	+	+	+	+	+	+	+	+	+	+	+
Edema	−	−	−	−	−	−	−	−	−	−	−	−	−	−	−	−	−	−	−
Mortality/death	−	−	−	−	−	−	−	−	−	−	−	−	−	−	−	−	−	−	−

*Note:* Positive (+) sign indicates normal, while negative (−) sign indicates no effect.

**TABLE 10:** Acute oral administration effect of methanol extracts of leaves and stem barks of *Osyris quadripartita* and *Toddalia asiatica* on the body weight (wt) of mice.

Tested samples	Dose (mg/kg)	Initial body wt at Day 0	Body wt at Day 7	Final body wt at Day 14	Body wt gain/loss
Water (negative control)	Distilled water	30.5 ± 0.37	33.0 ± 0.26	33.5 ± 0.31	3.0 ± 0.34

*Osyris quadripartita* leaf extract	1000	31.3 ± 0.08	30.3 ± 0.05	32.8 ± 0.12	1.5 ± 0.12
	1500	31.2 ± 0.14	30.3 ± 0.08	32.8 ± 0.18	1.6 ± 0.11
	2000	30.3 ± 0.22	29.3 ± 0.25	31.6 ± 0.32	1.3 ± 0.32

*Osyris quadripartita* stem bark extract	1000	32.5 ± 0.32	31.2 ± 0.20	33.8 ± 0.34	1.3 ± 0.22
	1500	31.0 ± 0.42	29.6 ± 0.34	32.2 ± 0.44	1.2 ± 0.32
	2000	31.8 ± 0.08	30.8 ± 0.09	33.2 ± 0.12	1.4 ± 0.06

*Toddalia asiatica* leaf extract	1000	32.0 ± 0.11	31.0 ± 0.12	33.8 ± 0.21	1.8 ± 0.22
	1500	32.3 ± 0.34	31.3 ± 0.27	34.0 ± 0.18	1.7 ± 0.32
	2000	32.3 ± 0.36	31.3 ± 0.32	34.1 ± 0.41	1.8 ± 0.42

*Toddalia asiatica* stem bark extract	1000	32.2 ± 0.14	31.2 ± 0.11	33.8 ± 0.17	1.6 ± 0.20
	1500	31.0 ± 0.23	30.0 ± 0.18	33.0 ± 0.24	2.0 ± 0.26
	2000	32.3 ± 0.30	30.3 ± 0.31	32.8 ± 0.34	0.5 ± 0.43^∗^

^∗^indicates significantly different values (*p* < 0.05) compared to the control (distilled water).

**TABLE 11:** Acute oral administration effect of chloroform extracts of leaves and stem barks of *Osyris quadripartita* and *Toddalia asiatica* on the body weight (wt) of mice.

Tested samples	Dose (mg/kg)	Initial body wt at Day 0	Body wt at Day 7	Final body wt at Day 14	Body wt gain/loss
Water (negative control)	Distilled water	30.5 ± 0.37	33.0 ± 0.26	33.5 ± 0.31	3.0 ± 0.34

*Osyris quadripartita* leaf extract	1000	31.6 ± 0.31	30.6 ± 0.33	33.4 ± 0.28	1.8 ± 0.24
	1500	31.0 ± 0.28	30.0 ± 0.26	32.6 ± 0.29	1.6 ± 0.25
	2000	30.8 ± 0.44	29.8 ± 0.42	31.8 ± 0.36	1.0 ± 0.34

*Osyris quadripartita* stem bark extract	1000	31.8 ± 0.07	31.0 ± 0.12	32.8 ± 0.14	1.0 ± 0.21
	1500	32.0 ± 0.35	31.0 ± 0.28	32.9 ± 0.32	0.9 ± 0.33
	2000	32.4 ± 0.50	31.6 ± 0.52	32.8 ± 0.46	0.4 ± 0.42^∗^

*Toddalia asiatica* leaf extract	1000	32.6 ± 0.25	31.6 ± 0.28	33.8 ± 0.24	1.2 ± 0.24
	1500	30.3 ± 0.33	29.5 ± 0.31	31.3 ± 0.34	1.0 ± 0.26
	2000	32.2 ± 0.53	31.2 ± 0.41	33.6 ± 0.52	1.4 ± 0.48

*Toddalia asiatica* stem bark extract	1000	32.2 ± 0.13	31.2 ± 0.16	33.2 ± 0.18	1.0 ± 0.14
	1500	32.5 ± 0.13	31.5 ± 0.12	33.2 ± 0.12	0.7 ± 0.14^∗^
	2000	30.5 ± 0.41	29.6 ± 0.35	31.4 ± 0.36	0.9 ± 0.28^∗^

^∗^Indicates significantly different values (*p* < 0.05) compared to the control (distilled water).

The toxicity assessment revealed that mice treated with the studied extracts exhibited no significant changes in body weight (*p* > 0.05) within half an hour and up to 14 days post‐treatment. Exceptions were observed at higher doses, such as the methanol extract (2000 mg/kg) and chloroform extracts (1500 and 2000 mg/kg) of the stem bark of *T. asiatica*, as well as the chloroform extract (2000 mg/kg) of the stem bark of *O. quadripartita*, which produced significant changes (*p* < 0.05) (Tables 10 and 11). Studies on aqueous, methanol, and chloroform crude extracts of *O. quadripartita* [51], as well as the dichloromethane extract of *T. asiatica* leaves, reported no signs of toxicity or mortality in mice at doses of 2000 mg/kg body weight. However, extracts administered at higher concentrations caused significant reductions (*p* < 0.001) in head size, birth weight, and limb length in pregnant mice [52], indicating that such extracts should be used with caution during pregnancy. Similarly, the chloroform and methanol extracts tested in this study did not cause any changes in the body weights of the treated mice, indicating their nontoxic nature, as alterations in body weight are typical markers of toxic effects [53]. The present findings were generally in agreement with previous studies conducted on the acute toxicity effect of plant extract [54], and changes in body weights are considered indicators of the effects of various chemicals found in plant parts [55]. In general, the absence of mortality up to an oral dose of 2000 mg/kg suggests that the test extracts were safe, which could explain the routine use of these plants by local people for the traditional management of reported ailments. However, the dose progression and observation periods employed in this study did not strictly follow the sequential up‐and‐down procedure outlined in OECD Guideline 425, which may be considered a limitation.

## 4. Conclusion

The chloroform and methanol extracts of leaves and stem barks of *O. quadripartita* and *T. asiatica* generally showed weak‐to‐strong antimicrobial effects on the tested four bacterial and three fungal pathogens. Methanol stem Bark extract of *O. quadripartita* exhibited strong antibacterial activity against the methicillin‐resistant *S. aureus* with zone of inhibition value of 15.23 ± 0.25 mm at the highest concentration (175 mg/mL), while the same extract of the same part of both plants showed a moderate activity, at a minimum concentration of 100 mg/mL, against the same pathogen with inhibitory values of 10.67 ± 0.57 and 10.6 ± 0.11 mm, respectively. The methanol stem park extracts of *O. quadripartita* and *T. asiatica* also scored the lowest MIC values of 6.25 ± 00 and 12.5 ± 00 mg/mL against *S*. *aureus* and *S. pyogenes*, respectively. Additionally, the methanol leaf extract of *O. quadripartita* and stem barks of *T. asiatica* demonstrated MBC values of 25 and 50 mg/mL, respectively, against *S. pyogenes*. The phytochemical analysis confirmed the presence of tannins, alkaloids, saponins, flavonoids, and terpenoids in the aforementioned extracts of both parts of the studied plants, which was in line with the obtained weak‐to‐strong antimicrobial activity results. This study also indicated that methanol was found to be a better solvent than chloroform for both the extraction process and antimicrobial activity assay, which may be due to its amphiphilicity nature. The occurrence of major chemical constituents in the plants’ extracts is an indication that these and other solvent extracts, if properly fractionated using chromatographic techniques, could yield potential antimicrobial natural molecules. The chloroform and methanol extracts of the studied plant parts caused no mortality or indication of acute toxicity effect on the treated experimental mice up to a dose of 2000 mg/kg body weight both in 24 h and 14 days of observation period. This study supports the purported traditional medicinal usages of leaves and stems barks of *O. quadripartita* and *T. asiatica* and paves the way for further exploration of potential antimicrobial natural compounds. But further toxicity studies are needed to assure their safety in humans, quantify the major class of phytochemicals, and identify individual compounds using chromatographic techniques and advanced instruments, including FT‐IR, LC/MS, and NMR. Moreover, the dose progression and observation periods applied during the acute oral toxicity assessment in this study did not strictly adhere to the sequential up‐and‐down procedure outlined in OECD Guideline 425, which may be regarded as a limitation.

## Funding

The work was supported by the Office of Vice President for Research and Community Engagement, Haramaya University, Ethiopia (Grant No. HUSS_2018_06_01_26).

## Ethics Statement

This study was conducted after the necessary ethical clearance (Reference No. SBBT/20019/2026) was obtained from School Research Ethics Committee, School of Biological Science and Biotechnology, Haramaya University. The living area for mice allowed them to satisfy their basic needs, including the ability to eat, drink, urinate, and defecate and also regulate their body temperatures.

## Conflicts of Interest

The authors declare no conflicts of interest.

## Data Availability

All generated data are presented in the article.
